# Corrigendum: Iowa gambling task: there is more to consider than long-term outcome. Using a linear equation model to disentangle the impact of outcome and frequency of gains and losses

**DOI:** 10.3389/fnins.2013.00090

**Published:** 2013-06-04

**Authors:** Annette Horstmann, Arno Villringer, Jane Neumann

**Affiliations:** ^1^Department of Neurology, Max Planck Institute for Human Cognitive and Brain Sciences, Max Planck SocietyLeipzig, Germany; ^2^Integrated Research and Treatment Center Adiposity Diseases, Leipzig University Medical CenterLeipzig, Germany; ^3^Clinic for Cognitive Neurology, University Hospital LeipzigLeipzig, Germany; ^4^Mind and Brain Institute, Berlin School of Mind and Brain, Humboldt University BerlinBerlin, Germany

A mistake regarding the exemplary weights given in Table 4 of our original publication was recently brought to our attention. We corrected the weights and give the complete and updated table in this commentary.

**Table 4:** **(corrected): Example (one subject's) least squares solution of the linear equation model for mean choices in block 5**.

**Deck**	**Long-term outcome (*x*_1_)**	**Gain frequency (*x*_2_)**	**Loss frequency (*x*_3_)**	**Choices [%]**
A	−0.86	−0.86	−1.47	0.15
B	−0.86	0.86	0.34	0.50
C	0.86	−0.86	0.79	0.10
D	0.86	0.86	0.34	0.25
Weights	−0.145	0.110	0.089

